# Cerebellar and brainstem differences in children with developmental coordination disorder: A voxel-based morphometry study

**DOI:** 10.3389/fnhum.2022.921505

**Published:** 2022-07-28

**Authors:** Kamaldeep K. Gill, Donna Lang, Jill G. Zwicker

**Affiliations:** ^1^Department of Rehabilitation Sciences, The University of British Columbia, Vancouver, BC, Canada; ^2^Brain, Behaviour, and Development Theme, British Columbia Children’s Hospital Research Institute, Vancouver, BC, Canada; ^3^Department of Radiology, The University of British Columbia, Vancouver, BC, Canada; ^4^Department of Occupational Science and Occupational Therapy, The University of British Columbia, Vancouver, BC, Canada; ^5^Department of Pediatrics, The University of British Columbia, Vancouver, BC, Canada

**Keywords:** developmental coordination disorder, motor skills disorder, cerebellum, voxel-based morphometry, grey matter

## Abstract

Developmental coordination disorder (DCD) is a neurodevelopmental disorder that significantly impairs a child’s ability to learn motor skills and to perform everyday activities. The cause of DCD is unknown; however, evidence suggests that children with DCD have altered brain structure and function. While the cerebellum has been hypothesised to be involved in developmental coordination disorder, no studies have specifically examined cerebellar structure in this population. The purpose of our study was to examine cerebellar differences in children with DCD compared to typically-developing children. Using voxel-based morphometry, we assessed cerebellar morphology in children 8–12 years of age. Forty-six children (12 typically-developing and 34 with DCD) were investigated using high resolution T1-weighted images, which were then processed using the spatially unbiased atlas template of the cerebellum and brainstem (SUIT) toolbox for a region of interest-based examination of the cerebellum. Results revealed that children with DCD had reduced grey matter volume in several regions, namely: the brainstem, right/left crus I, right crus II, left VI, right VIIb, and right VIIIa lobules. Further, Pearson correlations revealed significant positive associations between the total motor percentile score on the Movement Assessment Battery for Children-2 and regions that had reduced grey matter volume in our cohort (brainstem, left crus I, right VIIb, and right VIIIa). These findings indicate that reductions in cerebellar grey matter volume are associated with poorer motor skills. Given the cerebellum’s involvement in internal models of movement, results of this study may help to explain why children with DCD struggle to learn motor skills.

## Introduction

Developmental Coordination Disorder (DCD) is a neurodevelopmental disorder that affects ∼450,000 Canadian children, or roughly 1-to-2 children in every classroom (American Psychiatric Association, 2013; Statistics Canada, 2020). DCD significantly impairs a child’s ability to learn motor skills and to perform everyday activities, such as getting dressed, tying shoelaces, or riding a bicycle (Zwicker et al., 2012). As a result of motor skill impairments, children with DCD have poorer academic achievement, and reduced participation in self-care, social, and leisure activities (Zwicker et al., 2013; Izadi-Najafabadi et al., 2019). It was once believed that children with DCD would outgrow their motor difficulties; however, evidence suggests that difficulties persist into adolescence and adulthood (Kirby et al., 2014).

Although the characteristics of DCD suggest potential neural correlates may be involved, the cause of DCD is unknown, and the role of the brain has only recently been investigated through neuroimaging studies (Biotteau et al., 2016; Brown-Lum and Zwicker, 2017). It is posited that DCD may be related to the central nervous system pathology, particularly the parietal lobe, basal ganglia, and the cerebellum (Zwicker et al., 2009, 2010; Peters et al., 2013). Given its role in motor control, cognition, language and emotional processing, a large body of evidence suggests cerebellar deficits contribute to the sequelae of DCD (Zwicker et al., 2009; Peters et al., 2013; Brown-Lum and Zwicker, 2015; Biotteau et al., 2016).

The cerebellum is a complex neurological structure that contains more than half of the brain’s total number of neurons (Kandel, 2013). Anatomically, the cerebellum is divided into three lobes and ten lobules: anterior lobe (lobules I–V), posterior lobe (lobules VI–IX), and flocculonodular lobe (lobule X) (Stoodley et al., 2012). Within the cerebellum, different regions are involved in motor control vs. cognitive and emotional processing. The functional topography of the human cerebellum is based on anatomical connections with the cerebral cortex and the spinal cord. Lobules I–V and lobule VIII are predominately involved in sensorimotor functioning. Lobules VI and VII are functionally connected with the frontal and parietal association cortices and engage in cognitive functioning. Lobule IX is thought to be involved in multiple cortical networks, including the default mode network. Lobule X is known as the vestibulocerebellum, as it receives vestibular and visual information and is involved in balance, vestibular reflexes, and eye movements (Stoodley et al., 2012).

Cerebellar functional topography is important when considering its involvement in developmental disorders (Stoodley, 2014, 2016). Cerebellar abnormalities have been consistently observed in a number of neurodevelopmental disabilities, such as autism spectrum disorder (ASD), attention deficit hyperactivity disorder (ADHD), learning disabilities (LD), and DCD (Zwicker et al., 2009, 2011; Stoodley, 2014). The cerebellum is thought to be one of the last brain structures to fully develop (Stoodley, 2016). Relative to other brain regions, the cerebellum undergoes enormous growth between 24- and 40-weeks post-conception, increasing 5-fold in volume and 30-fold in surface area (D’Mello et al., 2015). This substantial prenatal growth continues postnatally and makes the cerebellum especially vulnerable to developmental disruptions and damage. Data from clinical populations suggest that early cerebellar damage is associated with range of motor, cognitive, and affective outcomes in a location-dependent manner (Stoodley and Limperopoulos, 2016). Many neuroimaging studies have investigated the involvement of the cerebellum in neurodevelopmental disabilities in the paediatric population to examine potential early cerebellar damage. Recently, Stoodley (2014) found distinct patterns of cerebellar deficits that characterise ASD, ADHD, and LD. Specifically, children with ASD have reduced grey matter volume in lobules VIIIb, IX, and right crus I; children with ADHD show reduced grey matter volume in lobule IX; and children with dyslexia show decreased grey matter in left lobule VI (Stoodley, 2014).

Abnormalities of the cerebellum have been proposed as the main underlying mechanism that give rise to DCD due to its role in motor coordination and motor learning. To our knowledge, this is the first study to specifically examine the cerebellum in DCD. The purpose of this study was to investigate the cerebellar structural differences using voxel-based morphometry in children with DCD compared to typically-developing peers, and to correlate cerebellar regional grey matter volumes with clinical measures.

## Materials and methods

### Study design

This cross-sectional study evaluated differences in cerebellar morphology between children with DCD and typically-developing (TD) children. This study is part of a larger randomised control trial in which an intervention effect will be investigated (ClinicalTrials.gov ID: NCT02597751). The sample size calculation was calculated for the larger study, which yielded a sample of 25 participants per group (DCD and TD) to allow for a power of 80%, standard deviation of 2.5 and type one error of 0.05, to detect a clinically significant differences using *t*-tests for the main motor outcome measure. We aimed to recruit a target sample size of 30 per group to accommodate power calculation for neuroimaging measures.

This study was approved by the University of British Columbia/Children’s and Women’s Clinical Research Ethics Board. Parents/legal guardians provided informed written consent and children assented to participate in the study.

### Participants

Using sample of convenience, we recruited 8–12-year-old children with DCD from Dr. Zwicker’s research-integrated DCD Clinic at Sunny Hill Health Centre for Children, BC Children’s Hospital ADHD Clinic, and from the community in the Greater Vancouver area from September 2014 to January 2019. Typically-developing children were recruited from the community using bulletin boards at BC Children’s Hospital, UBC, and Vancouver Schools. The inclusion criteria for children with DCD were based on the four diagnostic criteria for DCD in the Diagnostic and Statistical Manual—5th edition (American Psychiatric Association, 2013) and international DCD guidelines (Blank et al., 2019): (1) total score ≤ 16th percentile on the Movement Assessment Battery for Children–2nd edition (MABC-2) (Henderson et al., 2007) (Criterion A—*motor skills below the expected level for age*); (2) a score in the suspected or indicative range on the DCD Questionnaire (DCDQ) (Wilson et al., 2009) (Criterion B—*motor skills deficit significantly interferes with activities of daily living, school performance, and leisure activities*); (3) parent-reported motor difficulties from a young age (Criterion C—*onset of symptoms in the early developmental period*); and (4) no other medical condition that could explain motor difficulties as per parent-report, clinical reports and/or medical examination (Criterion D—*excludes intellectual disability, visual impairment, or neurological condition, such as cerebral palsy*). Typically-developing children had no history of motor difficulties and a score ≥ 25th percentile on the MABC-2. Participants were excluded if they were born preterm (gestational age < 37 weeks) or diagnosed with any other neurodevelopmental disability (other than commonly co-occurring ADHD).

**FIGURE 1:**
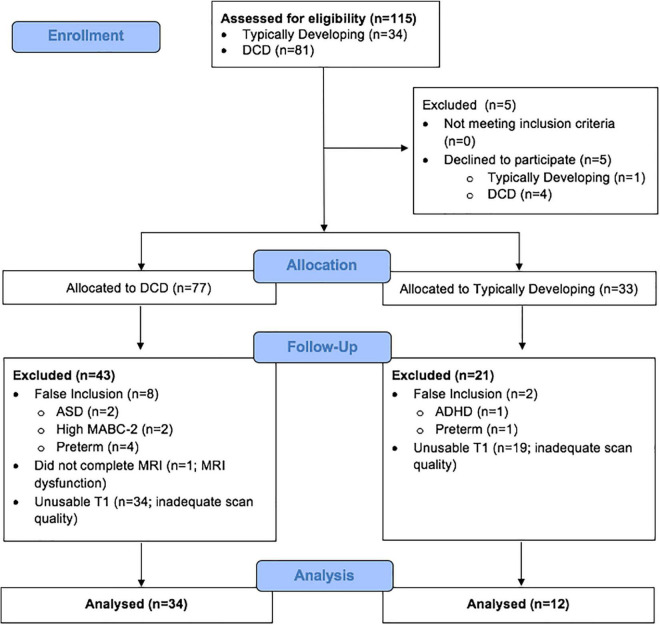
Flow diagram of participant inclusion/exclusion for voxel-based morphometry analysis.

This study included 115 children (TD = 34; DCD = 81) from whom 69 were excluded because they either declined to participate (*N* = 5), were missing T1 scan (*N* = 1), had co-morbidities of ASD or preterm birth (*N* = 10), or had insufficient data quality for VBM analysis (*N* = 53) (Figure 1). Our final sample was comprised of 46 children - 35 males (76%) and 11 females (24%); of this, 34 children were included in the DCD group and 12 were in the TD group (Table 1). All children participated in an MRI safety screening and an MRI simulator session to familiarise themselves with the MRI environment.

**TABLE 1:** Description of cohort (*N* = 46).

Clinical characteristics	DCD (*N* = 34) *N* (%) or mean (SD)	TD (*N* = 12) *N* (%) or mean (SD)	*P*-value
Male	28 (82.4)	7 (58.3)	0.16
Age at MRI (years)	10.2 (1.6)	10.0 (1.5)	0.79
MABC-2 (percentile)	6.6 (8.9)	59.7 (25.1)	<0.001
Conners ADHD Index (*t*-scores)	85.7 (8.3)	54.8 (11.7)	<0.001
Total intra-cranial volume (L)	1.51 (0.1)	1.52 (0.1)	0.79

ADHD, attention deficit hyperactivity disorder; DCD, developmental coordination disorder; MABC-2, Movement Assessment Battery for Children–2nd ed.; TD, typically- developing children.

### Behavioural measures

#### Movement Assessment Battery for Children–2nd edition

The MABC-2 is a standardised and norm-referenced test that assesses a child’s performance on a series of gross and fine motor tasks, which are scored and rated in three areas of motor performance: (1) manual dexterity; (2) aiming and catching; and (3) balance (Henderson et al., 2007). The MABC-2 is considered to be a valid and reliable measure to assess motor impairments in children and has an internal consistency of α = 0.90 (Brown and Lalor, 2009). In this study, the cut-off score of at or below the 16th percentile for the total MABC-2 score was used to determine the DCD group as per international DCD guidelines (Blank et al., 2019). Children who scored at or above the 25th percentile were classified as typically-developing.

#### Developmental Coordination Disorder Questionnaire

The DCDQ is a parent-rated questionnaire that is used as a screening tool to identify functional motor difficulties in children 5–15 years old (Wilson et al., 2009). Parents compare their child’s performance in various everyday tasks to the performance of their peers. The DCDQ consists of 15 activities which are rated on a 5-point scale and grouped into three different categories: (1) control during movement; (2) fine motor/handwriting; and (3) general coordination. Scores are summed to a total score between 15 and 75 and categorised as “indicative of DCD,” “suspect DCD,” or “probably not DCD” depending on the child’s age; higher scores indicate better motor coordination (Wilson et al., 2009). The DCDQ has high internal consistency (α = 0.94) and adequate sensitivity (85%) (Wilson et al., 2009). This questionnaire has good reliability and validity; therefore, it is a recommended screening tool in the international guidelines for DCD (Blank et al., 2019).

#### Conners 3 Attention Deficit Hyperactivity Disorder Index

The Conners 3 ADHD Index (Conners 3 AI) parent form was used to assess for ADHD symptoms (Conners et al., 2009). This short-form questionnaire can distinguish between a child that does or does not have ADHD. This is a norm-referenced assessment that is based on a large North American sample. It is one of the most commonly used screening tools to assess for ADHD symptoms in both research and clinical settings (Conners et al., 2009). The Conners 3 ADHD Index has high internal consistency (α = 0.90), high predicative validity, mean test-retest reliability of 0.87, and inter-rater agreement of 0.75 (Morales-Hidalgo et al., 2017). A score above 70 is considered in the clinically significant range for ADHD symptomatology. For the purpose of this study, the Conners 3 ADHD Index was used to quantify the degree of attentional difficulties. Children with DCD are more likely than typically-developing children with have attentional difficulties, with over 50% of children with DCD having a co-occurring ADHD diagnosis (Dewey et al., 2002; Fliers et al., 2010).

### Neuroimaging measures

#### Image acquisition

For each participant, high resolution isotropic T1-weighted 3D scans were acquired on a 3-Tesla General-Electric Discovery MR750 MRI scanner using a 32-channel head coil. The high-resolution T1-weighted image was acquired with the following parameters: 3D SPGR, echo time = 30 ms, repetition time = 3,000 ms, FOV = 256, matrix size = 256 × 256, flip angle = 12°, number of slices = 256, slice thickness = 1 mm, interleaved with no gaps (voxel size 0.9375 mm × 0.9375 mm × 1 mm). Scans with significant motion artefact or poor grey/white matter differentiation were excluded from the larger sample to produce the current dataset. Specifically, all scans were visually inspected for motion-related artefacts, such as blurring, ghosting, and stripping (Reuter et al., 2015). Further, image quality was assessed for head coverage, wrapping artefact, radiofrequency noise, signal inhomogeneity, susceptibility artefact, and ringing artefact (Reuter et al., 2015). An ordinal score was given to each image based on motion artefacts and image quality (pass, questionable, or fail) using standardised methodology (Harvard Center for Brain Science Center for Brain Science, 2020). Only scans that passed the final quality check were included in the analysis.

#### Voxel-based morphometry

The MR images were processed using VBM, a computational technique that measures differences in regional grey matter density and/or volume through a voxel-wise comparison (Ashburner and Friston, 2000). We used the SUIT (Spatially Unbiased Atlas Image Template) toolbox through SPM12 in MATLAB 2016a, which allows for a ROI-based examination of the cerebellum using a high-resolution atlas and template (Diedrichsen, 2006). The more commonly used Montreal Neurological Institute (MNI) template provides little contrast for the cerebellum, while the SUIT template preserves the anatomical details of the cerebellum and allows for better localisation of cerebellar findings (Diedrichsen, 2006). SUIT provides an excellent template for measuring specific cerebellar lobular findings and may be more statistically powerful than whole brain VBM approaches, providing more power for subtle group differences in cerebellar grey matter between children with DCD and typically-developing children (Diedrichsen, 2006).

Initially, a quality check was conducted to ensure scans were of adequate quality for the analysis (good signal-to-noise ratio and grey/white matter contrast). Then, the origin of each scan was set to the anterior commissure to normalise all the images to the same stereotactic space. The cerebellum was then isolated from the T1 images and segmented into grey matter, white matter, and cerebrospinal fluid. Next, using the Diffeomorphic Image Registration Algorithm (DARTEL), the segmented images were normalised into the SUIT atlas template—a high-resolution template of the human cerebellum based on the anatomy of 20 healthy individuals—to account for global brain shape differences between participants. Lastly, using affine transformation, the images were re-sliced into SUIT space using DARTEL with standard smoothing at 4 mm full-width-half-maximum (FWHM) Gaussian kernel. The smoothed, modulated, normalised data were used in the statistical analysis. The modulation step involved scaling by the amount of contraction or expansion that took place in the normalisation step. This ensured that the total amount of grey matter remained the same as it would in the original images. This step is recommended since we are interested in volume changes rather than concentration differences in grey matter (Good et al., 2001; Mechelli et al., 2005).

The SUIT probabilistic atlas is a grey matter template only and excludes white matter. This process results in 28 grey matter volume measurements (clusters of 1 mm × 1 mm × 1 mm voxels) reflecting the ten bilateral lobules (I-X right and I-X left lobules; lobules I-IV are combined into one measure and lobule VII is divided into VIIB, crus I, and Crus II; lobule VIII is divided into VIIIa and VIIIb) and vermis VI-X (Diedrichsen, 2006).

### Data analysis

#### Participant characteristics

SPSS Statistics software package version 25.0 was used to analyze behavioural data. To compare the sex distribution between groups, we used the Chi-square test. To compare differences in age, total intracranial volume (TIV), MABC-2 scores, and Conners 3 ADHD Index scores, we used two-tail student’s *t*-tests with a significance level of *p* < 0.05. Due to the unequal group sizes of DCD and TD, a Levene’s test was performed to ensure the assumption of homogeneity of variance was met.

#### Voxel-based morphometry

The statistical comparison between the DCD and TD groups was performed using PALM–Permutation Analysis of Linear Models (Winkler et al., 2016). PALM allows for statistical inferences of imaging data using permutations methods that do not require the assumptions of parametric analysis. This analysis used Threshold Free Cluster Enhancement (TFCE), a voxel-wise statistical method in which each voxel’s value represents the cluster-like spatial support in accordance with the spatial neighbourhood information (Winkler et al., 2014). TFCE estimates a voxel value that represents the accumulative cluster-like local spatial support at a range of cluster-forming thresholds (Winkler et al., 2014). TFCE does not enforce assumptions of cluster size; thus, improving the results’ stability compared to cluster thresholding (Winkler et al., 2014). To assess for group differences in grey matter, a two-sample *t*-test was performed. The VBM toolbox of Statistical Parametric Mapping was used with default parameters to segment the voxels of T1-weighted brain volume into four classes: white matter (WM), grey matter (GM), and cerebrospinal fluid (CSF). WM, GM, and CSF volumes were summed to provide an estimate of total intracranial volume (TIV). TIV was demeaned and used as a nuisance variable/covariate in the analysis. To assess the correlation between the total percentile MABC-2 scores and grey matter volume, Pearson correlation was used. The magnitude of each correlation was interpreted to characterise the strength of the correlation: *r* = 0.00–0.25 indicated little correlation, if any; 0.26–0.49 indicated low correlation; 0.50–0.69 indicated moderate correlation, 0.70–0.89 indicated high correlation, and 0.90–1.00 indicated very high correlation (Cohen, 1992). An alpha of 0.01 was chosen to minimise type 1 errors. All results are reported with TFCE correction; however, results are uncorrected for multiple comparisons over contrasts due to the small sample size. Results are presented at *p* < 0.01 with cluster size thresholded at 50 voxels, which is comparable to previous publications of VBM and neurodevelopmental disabilities (D’Mello et al., 2015).

## Results

### Participant characteristics

Table 1 presents demographic and behavioural characteristics of the sample. The DCD group included 34 participants, 31 of whom had elevated Conners *t*-scores (≥70); almost two-thirds of the sample of children with DCD (21/34) had a co-occurring diagnosis of ADHD. The TD group included 12 participants. There were no group differences in age (*p* = 0.79), sex (*p* = 0.16), or total intra-cranial volume (*p* = 0.79). There was a higher number of male participants in the DCD group, as DCD is more common in males compared to females (ranging from 2:1 male: female ratio in some studies to 7:1 male: female ratio in other studies) (Kadesjö and Gillberg, 1998; Lingam et al., 2009; American Psychiatric Association, 2013). There was also a significant difference in MABC-2 total percentile scores and Conners ADHD Index t-scores (*p* < 0.001) between the DCD and TD groups, which is similar to the co-occurrence rate reported in the literature (Lingam et al., 2009).

### Cerebellar analyses

#### Group differences in grey matter

**FIGURE 2:**
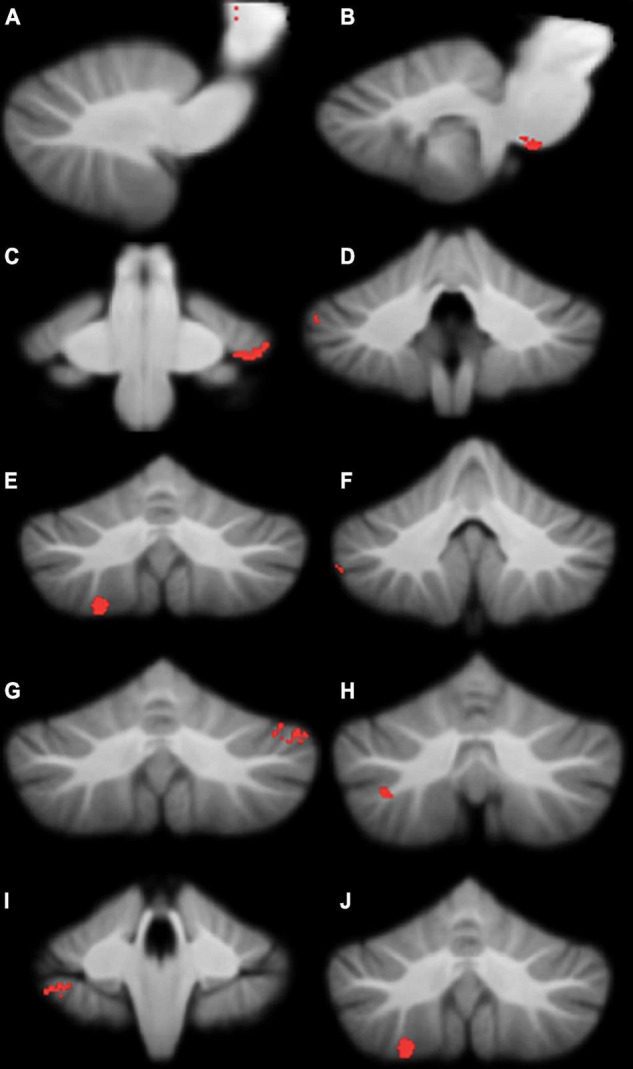
Significant grey matter reductions in children with developmental coordination disorder (DCD) in comparison to typically-developing children. **(A)** brainstem; **(B)** brainstem; **(C)** left lobule VI; **(D)** right crus I; **(E)** right crus I; **(F)** right crus I; **(G)** left crus I; **(H)** right crus II; **(I)** right lobule VIIb; **(J)** right lobule VIIIa. All results reported with threshold free cluster enhancement (TFCE) correction, uncorrected across contrast at *p* < 0.01; cluster size threshold at 50 voxels.

Compared to typically-developing children, children with DCD had significantly reduced grey matter in the: (1) brainstem; cerebellar (2) right crus I/II; (3) left crus I; (4) left VI; (5) right VIIb; and (6) right VIIIa lobules (Table 2 and Figure 2). There were no cerebellar regions where children with DCD had greater grey matter volume compared to typically-developing children.

**TABLE 2:** Montreal Neurological Institute (MNI) coordinates for significant grey matter volume reductions in children with developmental coordination disorder compared to typically-developing children.

Location	*X*	*Y*	*Z*	*t*	Cluster size
Brainstem (A)	15	−21	0	2.74	63
Brainstem (B)	10	−31	−43	2.91	56
Left VI	−31	−35	−34	2.86	195
Right crus I (A)	48	−46	−35	3.12	127
Right crus I (B)	32	−86	−28	2.68	95
Right crus I (C)	51	−50	−44	2.80	68
Left crus I	−43	−61	−29	3.22	904
Right crus II	35	−64	−46	2.50	120
Right VIIb	39	−41	−46	3.23	83
Right VIIIa	25	−61	−55	3.69	1,554

This table refers to the clusters presented in Figure 2.

#### Motor function and regional cerebellar grey matter

**FIGURE 3:**
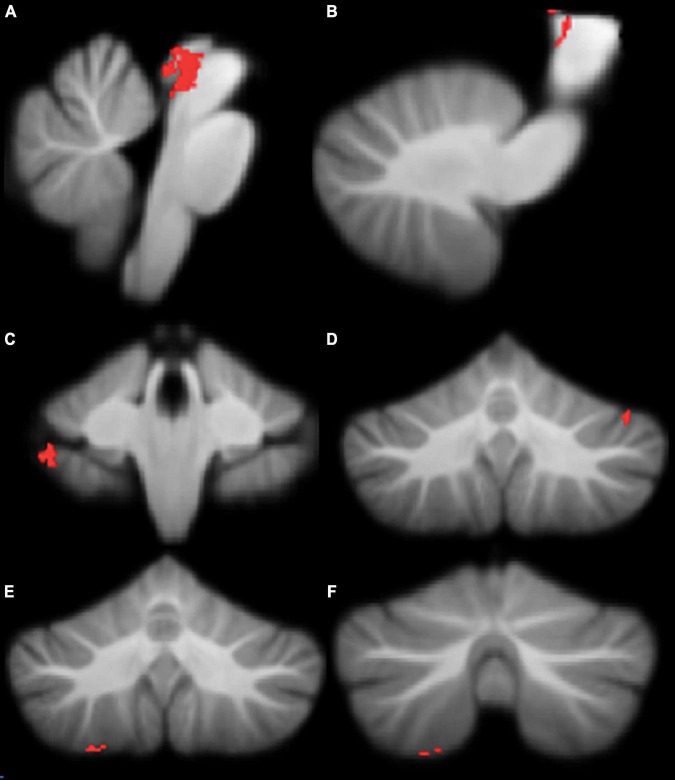
Significant positive correlations between grey Movement Assessment Battery for Children-2 (MABC-2) total percentile scores. **(A)** brainstem; **(B)** brainstem; **(C)** right lobule VIIb; **(D)** left crus I; **(E)** right lobule VIIIa; **(F)** right lobule VIIIa. All results reported with TFCE correction, uncorrected across contrast at *p* < 0.01; cluster size threshold at 50 voxels.

Motor functioning, measured by MABC-2 percentile score, was positively correlated with regional grey matter volume in the brainstem, left crus I, right VIIb, and right VIIIa lobules, in our cohort (Table 3 and Figure 3). Lower MABC-2 scores were related to smaller grey matter volume. No correlations of cerebellar grey matter with DCDQ scores met the 50-voxel threshold.

**TABLE 3:** Montreal Neurological Institute (MNI) coordinates for correlations between regional grey matter volumes and MABC-2 percentile scores.

Location	*X*	*Y*	*Z*	*r*	Cluster size
Brainstem (A)	2	−34	−6	0.99	639
Brainstem (B)	15	−24	−1	0.25	170
Brainstem (C)	−16	−23	−2	0.47	53
Left crus I	−41	−59	−26	0.97	70
Right VIIb	40	−40	−41	0.98	99
Right VIIIa	25	−60	−61	0.90	58
Right VIIIa	22	−70	−59	0.97	53

This table refers to the clusters presented in Figure 3.

#### Attention deficit hyperactivity disorder symptoms and regional cerebellar grey matter

**FIGURE 4:**
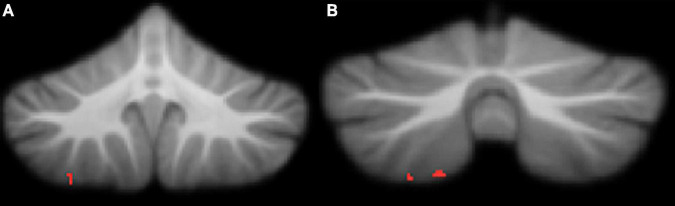
Significant negative correlations between grey matter and Conners ADHD Index *t*-scores. **(A)** right lobule VIIIa; **(B)** right lobule VIIIa. All results reported with threshold free cluster enhancement (TFCE) correction, uncorrected across contrast at *p* < 0.01; cluster size threshold at 50 voxels.

Attention deficit hyperactivity disorder symptomology in our cohort, measured by Conners ADHD Index *t*-score, was negatively correlated with regional grey matter volume in the right VIIIa lobule (Table 4 and Figure 4). Higher Conners scores were related to smaller grey matter volume.

**TABLE 4:** Montreal Neurological Institute (MNI) coordinates for correlations between regional grey matter volume and Conners ADHD Index *t*-scores.

Location	*X*	*Y*	*Z*	*r*	Cluster size
Right VIIIa	28	−56	−60	0.34	111
Right VIIIa	22	−69	−59	0.78	60

This table refers to the clusters presented in Figure 4.

## Discussion

We compared cerebellar regional grey matter in children with DCD and TD children and explored the relationship between cerebellar grey matter volume and clinical measures of motor skills and ADHD symptoms. To our knowledge, this is the first study to examine structural cerebellar differences in children with DCD using VBM. In summary, we found that, compared to typically-developing children, children with DCD had reduced grey matter volume in several regions, namely: the brainstem, right/left crus I, right crus II, left VI, right VIIb, and right VIIIa lobules. Further, MABC-2 scores of children with DCD were significantly and positively correlated with reduced grey matter volume in the brainstem, left crus I, right VIIb, and right VIIIa lobules. Further, we found that decreased grey matter volume was significantly correlated with poorer attentional skills in right VIIIa lobule. These findings indicate that reductions in cerebellar grey matter volume are associated with poorer motor and attentional skills. To interpret these results, we will review each region that seems to be implicated in DCD.

### Motor regions of the cerebellum

The brainstem, right and left crus I/II, and lobule VI are associated with motor functioning in the cerebellum (Stoodley et al., 2012). While no study has structurally analyzed the cerebellum in DCD, Zwicker et al. (2010) has been one of the few studies to report cerebellar findings using fMRI. They reported that children with DCD, compared to typically developing children, under-activated the right crus I/II, left lobule VI, and left Lobule IX when completing a motor accuracy task. The brainstem has not been previously investigated in the DCD population, hence, is an emergent finding. Each of these regions are discussed in detail in association with DCD symptomology.

#### Brainstem

Children with DCD had smaller grey matter volume in the brainstem compared to typically-developing children, and smaller volume in this region was associated with poorer motor skills. The brainstem is critical for motor function, autonomic regulation, and many neurocognitive functions (Lo et al., 2017). It undergoes rapid development during the third trimester of gestation and is particularly vulnerable to insults during this time (Lo et al., 2017). A disruption in the brainstem’s development, as evidenced in preterm birth, could lead to atypical development in higher order brain regions and associated behavioural impairments later in life (Dadalko and Travers, 2018). Brainstem underdevelopment and structural differences, particularly a decrease in grey matter volume, have been associated with both ASD and ADHD (Johnston et al., 2014; Dadalko and Travers, 2018). The brainstem is known to be involved in controlling locomotion and posture in association with the cerebellum (Drijkoningen et al., 2015). Given that children with DCD demonstrate altered activity in postural muscles (Johnston et al., 2002) and that the brainstem is known to be involved in controlling locomotion and posture in association with the cerebellum (Drijkoningen et al., 2015), our findings suggest that postural differences in children with DCD may be related to smaller brainstem volume.

#### Right crus I and II

The right crus I/II were reduced in grey matter volume in DCD compared to the typically-developing group. Right crus I/II are anatomically and functionally connected to several motor regions, including the prefrontal and parietal regions of the cerebral cortex (Hoover and Strick, 1999; Kelly and Strick, 2003; Strick et al., 2009; Buckner et al., 2011), both of which may be implicated in DCD. D’Mello et al. (2015) also reported right crus I/II grey matter reduction in children with ASD. These findings suggested that atypical structure of right crus I/II could result in under-connectivity with multiple cortical regions that are involved in imitation and praxis, leading to motor impairments in ASD (D’Mello et al., 2015). Given the high concordance of ASD and DCD, it is possible that reduced grey matter volume in right crus I/II could contribute to motor impairments in children with DCD, with or without other co-occurring conditions (Provost et al., 2007). Children with DCD have difficulties with visual processing, visuospatial navigation, decision-making, and motor performance, which may partially be attributed to reduced grey matter volume in right crus I/II (Wilmut et al., 2007; Asonitou et al., 2012; Chen et al., 2012).

#### Left crus I

We observed that children with DCD had smaller grey matter volume in left crus I compared to typically-developing children. Reduced grey matter volume in this region was significantly correlated with lower MABC-2 percentile scores, indicating a strong relationship between motor dysfunction and crus I grey matter volume. Previously, left crus I has been associated with working memory and executive functions, which are known to be adversely affected in DCD (Stoodley et al., 2012; Wilson et al., 2013; Leonard and Hill, 2015). Zwicker et al. (2011) also reported under-activation in the left crus I during motor learning in DCD. Adams et al. (2014) suggest that children with DCD struggle with motor learning due to an internal modelling deficit. Left crus I is seen to be involved in processes that require internal modelling, such as mental rotation and spatial transformation, and imitation and praxis, all of which are seen to be impaired in children with DCD (Stoodley and Schmahmann, 2009; Wilson et al., 2013; Reynolds et al., 2015b). Given the role of left crus I in cognitive functioning associated with motor learning (i.e., executive functioning, internal modelling), it is possible that the atypical morphology may be contributing to the motor impairments associated with DCD.

#### Left lobule VI

Children with DCD had reduced left lobule VI grey matter compared to typically-developing children. Additionally, reduced left lobule VI grey matter was associated with poorer motor scores on the MABC-2, suggesting that this region may be key to motor functioning.

Lobule VI is known to form part of the sensorimotor network of the cerebellum and left hemisphere has been linked to visuospatial processing (Habas et al., 2009; Stoodley and Schmahmann, 2009), which is known to be adversely affected in children with DCD (Wilson and McKenzie, 1998; Zwicker et al., 2009). Reduced grey matter volume in this region in children with DCD may account for difficulties in executive functions, attention, and motor processes, as lobule VI is functionally connected to the dorsal premotor cortex and the dorsolateral prefrontal cortex (Stoodley and Schmahmann, 2009; Bernard et al., 2012). Children with DCD have increased attentional and motor difficulties, which is in keeping with our volumetric findings in this study. Our current and other previously published findings suggest that lobule VI is critical for motor tasks and altered structure and function may be significant predictors for DCD symptomology (Zwicker et al., 2011; Biotteau et al., 2016; Debrabant et al., 2016).

### Cognitive regions of the cerebellum

Within the cerebellum, different regions are involved in motor control vs. cognitive and emotional processing. The functional topography of the human cerebellum is based on anatomical connections with the cerebral cortex and the spinal cord. Literature indicated that Lobules VII are functionally connected with the frontal and parietal association cortices and engage in cognitive functioning (Stoodley et al., 2012). Specifically, right lobule VIIb is associated with symptomology of DCD as follows.

#### Right lobule VIIb

Grey matter volume was reduced in the right lobule VIIb in children with DCD compared to TD children. There was also a strong, significant positive correlation between right lobule VIIb grey matter volume and motor functioning, with lower grey matter volume associated with poorer motor performance. Right lobule VIIb is involved in executive functioning tasks, complex decision making, emotional processing, and mental rotation, all of which are affected in children with DCD (Adolphs et al., 1996; Stoodley et al., 2012; Reynolds et al., 2015a; Bernardi et al., 2018). Given the role of lobule VIIb in cognitive functioning associated with motor learning (i.e., executive functioning, internal modelling), it is possible that the atypical morphology may be contributing to the motor impairments associated with DCD.

### Attentional regions of the cerebellum

Literature suggests that motor difficulties experienced by children with DCD may be partly due to impairments in self-regulation (e.g., monitoring performance) and emotional regulation (e.g., sustaining motivation, attentional regulation) (Blank et al., 2019). Similar to previous literature, our findings also indicate that motor impairments seen in children with DCD may be associated with attentional difficulties. Specifically, significant differences were seen in the right lobule VIIIa and described below in detail.

#### Right lobule VIIIa

Right lobule VIIIa was smaller in children with DCD and correlated with poorer motor skills and higher attentional difficulties in the same children. We observed that right lobule VIIIa was significantly and negatively correlated with attentional measures (Conners ADHD Index t-score), indicating that as decreased grey matter volume was associated with greater ADHD symptomology. Generally, lobule VIIIa receives input from the sensorimotor regions of the cerebral cortex and therefore is seen to be active during sensorimotor tasks, specifically requiring motor control (Stoodley et al., 2012). In healthy individuals, right lobule VIIIa is active during cognitive tasks, including verb generation, working memory paradigms, finger tapping tasks, and mental rotation, all of which are deficits in children with DCD (Alloway, 2012; Stoodley et al., 2012; Wilson et al., 2013; Adams et al., 2014; Reynolds et al., 2015b; Buszard et al., 2017; Bernardi et al., 2018). Specifically, lobule VIIIa is part of the cerebro-cerebellar loop which is important in working memory and the maintenance and storage of information (Habas et al., 2009). Working memory is closely related to attention and a prominent deficit associated with ADHD (Fassbender et al., 2011). As mentioned earlier, attentional difficulties are more prominent in children with DCD when compared to TD children; therefore, it is not surprising that structural differences in right lobule VIIIa are related to increased attentional difficulties (Fliers et al., 2010). Lobule VIIIa is responsible for sensorimotor processing and integration of cognitive resources in order to carry out motor skills, as evidenced by the significant and strong correlation with both motor and attentional severity and reduced grey matter volume in this region in children with DCD.

### Summary

Given the role of the cerebellum in motor coordination and motor learning, we previously hypothesised that the cerebellum was implicated in DCD (Zwicker et al., 2009). The current findings support and strengthen the role of the cerebellum in this disorder by showing that there were structural cerebellar deficits in DCD, and that these deficits were associated with poorer motor and attentional skills common in DCD. Considering the high rate of co-occurring ADHD in our sample, consistent with previous literature, it is important to consider the influence of attentional difficulties in driving our results. From a pharmacological perspective, the cerebellum is one of the main regions that shows altered activation following a single dose of methylphenidate (Fliers et al., 2010), suggesting that the cerebellar differences are functionally significant in terms of the behavioural profile of ADHD. The first quantitative study of brain morphometry in ADHD, reported smaller overall cerebellar volumes in children with ADHD relative to typically developing peers (Buckner et al., 2011). In one of the first longitudinal studies investigating the neurobiological underpinnings of ADHD, the differences in cerebellar volume persisted throughout development and correlated with symptoms severity of ADHD (Stoodley, 2014). There has been inconsistency with structural MRI findings in ADHD, with reports of reduced grey matter volume in the posterior vermal regions (lobules VII-X), left cerebellar lobules IV-VI, VIII, IX, and X and right cerebellar lobules IV, Crus I, VIII, and IX in children with ADHD relative to a typically developing comparison group (Fliers et al., 2010). Most consistently, grey matter reductions have been reported in the posterior cerebellum, specifically bilaterally in lobule IX, right lobule VIIIA, and posterior inferior vermis (VII-X) (Stoodley, 2016). In this current study, right lobule VIIIA emerged as a region that is correlated with attentional measures, which is consistent with previous literature. No other regions associated with ADHD, as described in literature, emerged which may further validate that the other findings in this study are specific to motor impairments experienced by children with DCD. However, it is important to note that attention and motor skills go hand in hand. It is known that attention and behavioural flexibility are needed for motor learning of functional motor tasks (Dewey et al., 2002).

Damage or developmental abnormalities affecting the cerebellum not only impede basic processing and functioning of the cerebellum, but also have further effects on cerebellar modulation of cerebro-cerebellar loops that may be relevant to DCD (D’Mello et al., 2015). For example, preterm infants—a group at high risk of DCD—who sustain a cerebellar injury often show impaired growth of the contralateral cerebral cortex along with impairments in expressive language, delayed receptive language, cognitive deficits, and motor impairments (Limperopoulos et al., 2010; Edwards et al., 2011).

In the current study, the degree of grey matter reduction in cerebellum subregions was significantly correlated with severity of motor impairments in children with DCD. This suggests the processing of motor and cognitive functions provided by the cerebellum is relevant to a range of DCD symptoms, such as motor skills, attention, and executive functioning. Our data serve to support this concept as cerebellar regions correlated with poorer motor functioning are associated with the fronto-parietal, sensorimotor, ventral attention, and default mode network, which have all been seen to be impaired in children with DCD (Zwicker et al., 2010; Thornton et al., 2018; Rinat et al., 2020). Grey matter abnormalities in cerebellum in children with DCD likely impact cerebral cortical networks that support both motor and non-motor functioning and contribute to DCD symptomology.

### Limitations

The findings presented here should be considered in the context of several strengths and limitations. First, while this study allows us to investigate the cerebellar involvement in DCD, the association of the regional grey matter volume to clinical symptoms and function is indirect. Further, the clinical measures with significant correlations were inconsistent with respect to their objective/subjective nature; motor assessments were standardised, whereas, attentional symptoms were parent-reported. This study is also a cross-sectional study which investigated the cerebellar structure at one given time point, not allowing for a causal inference. In the future, it would be of benefit to use a longitudinal cohort design. Second, as there are no standardised quantification guides to measure degree of motion artefact at this time, we relied on visual inspection by trained raters based on established guidelines (Reuter et al., 2015; Harvard Center for Brain Science Center for Brain Science, 2020). Due to the stringent nature of excluding scans with motion artefact or poor image quality, the final sample of our study was smaller than anticipated. This limitation prevented our group from comparing children with DCD to those that have co-occurring DCD and ADHD. Specifically, the typically developing group was smaller than anticipated. This may potentially lead to finding spurious group differences as well as lower power that limits detection of subtle differences that might otherwise be possible with larger samples. It is recommended that future studies consider examining the cerebellar differences in children with DCD and other co-occurring conditions, such as ADHD, ASD, and learning disabilities, as well as the longitudinal nature of cerebellar development in these groups.

## Conclusion

We investigated the cerebellar grey matter volume differences in children with DCD compared to typically developing children. Our results indicate that children with DCD have smaller grey matter volume in key motor and cognitive regions: the brainstem, right/left crus I, right crus II, left VI, right VIIb, right VIIIa lobules. Further, lower MABC-2 scores and higher Conners ADHD Index scores were associated with reduced grey matter volume. Given the cerebellum’s involvement in internal models of movement, results of this study may help to explain why children with DCD struggle to learn motor skills.

## Data availability statement

The raw data supporting the conclusions of this article will be made available by the authors, without undue reservation.

## Ethics statement

This study was approved by the University of British Columbia/Children’s and Women’s Clinical Research Ethics Board. Parents/legal guardians provided informed written consent and children assented to participate in the study.

## Author contributions

KG: investigation, data curation, methodology, validation, formal analysis, writing—original draft, and visualisation. DL: methodology, formal analysis, and writing—review and editing. JZ: conceptualisation, methodology, resources, writing—review and editing, supervision, project administration, and funding acquisition. All authors contributed to the article and approved the submitted version.
